# Predicting functional decline in aging and Alzheimer’s disease with PET-based Braak staging

**DOI:** 10.1093/braincomms/fcae043

**Published:** 2024-02-26

**Authors:** Arthur C Macedo, Joseph Therriault, Cécile Tissot, Jaime Fernandez-Arias, Pamela C L Ferreira, Paolo Vitali, Stijn Servaes, Nesrine Rahmouni, Marie Vermeiren, Gleb Bezgin, Firoza Z Lussier, Jenna Stevenson, Yi-Ting Wang, Kely Quispialaya Socualaya, Peter Kunach, Tahnia Nazneen, Seyyed Ali Hosseini, Vanessa Pallen, Alyssa Stevenson, João Pedro Ferrari-Souza, Bruna Bellaver, Douglas Teixeira Leffa, Kok Pin Ng, Eduardo R Zimmer, Tharick A Pascoal, Serge Gauthier, Pedro Rosa-Neto

**Affiliations:** Translational Neuroimaging Laboratory, The McGill University Research Centre for Studies in Aging, Douglas Mental Health Institute, Montreal, Quebec, H4H 1R3, Canada; Department of Neurology and Neurosurgery, McGill University, Montreal, Quebec, H3A 1A1, Canada; Montreal Neurological Institute, McGill University, Montreal, Quebec, H3A 2B4, Canada; Translational Neuroimaging Laboratory, The McGill University Research Centre for Studies in Aging, Douglas Mental Health Institute, Montreal, Quebec, H4H 1R3, Canada; Department of Neurology and Neurosurgery, McGill University, Montreal, Quebec, H3A 1A1, Canada; Montreal Neurological Institute, McGill University, Montreal, Quebec, H3A 2B4, Canada; Translational Neuroimaging Laboratory, The McGill University Research Centre for Studies in Aging, Douglas Mental Health Institute, Montreal, Quebec, H4H 1R3, Canada; Department of Neurology and Neurosurgery, McGill University, Montreal, Quebec, H3A 1A1, Canada; Montreal Neurological Institute, McGill University, Montreal, Quebec, H3A 2B4, Canada; Translational Neuroimaging Laboratory, The McGill University Research Centre for Studies in Aging, Douglas Mental Health Institute, Montreal, Quebec, H4H 1R3, Canada; Department of Neurology and Neurosurgery, McGill University, Montreal, Quebec, H3A 1A1, Canada; Montreal Neurological Institute, McGill University, Montreal, Quebec, H3A 2B4, Canada; Department of Psychiatry, School of Medicine, University of Pittsburgh, Pittsburgh, PA, 15213, USA; Translational Neuroimaging Laboratory, The McGill University Research Centre for Studies in Aging, Douglas Mental Health Institute, Montreal, Quebec, H4H 1R3, Canada; Department of Neurology and Neurosurgery, McGill University, Montreal, Quebec, H3A 1A1, Canada; Montreal Neurological Institute, McGill University, Montreal, Quebec, H3A 2B4, Canada; Translational Neuroimaging Laboratory, The McGill University Research Centre for Studies in Aging, Douglas Mental Health Institute, Montreal, Quebec, H4H 1R3, Canada; Department of Neurology and Neurosurgery, McGill University, Montreal, Quebec, H3A 1A1, Canada; Montreal Neurological Institute, McGill University, Montreal, Quebec, H3A 2B4, Canada; Translational Neuroimaging Laboratory, The McGill University Research Centre for Studies in Aging, Douglas Mental Health Institute, Montreal, Quebec, H4H 1R3, Canada; Department of Neurology and Neurosurgery, McGill University, Montreal, Quebec, H3A 1A1, Canada; Montreal Neurological Institute, McGill University, Montreal, Quebec, H3A 2B4, Canada; Translational Neuroimaging Laboratory, The McGill University Research Centre for Studies in Aging, Douglas Mental Health Institute, Montreal, Quebec, H4H 1R3, Canada; Translational Neuroimaging Laboratory, The McGill University Research Centre for Studies in Aging, Douglas Mental Health Institute, Montreal, Quebec, H4H 1R3, Canada; Department of Neurology and Neurosurgery, McGill University, Montreal, Quebec, H3A 1A1, Canada; Montreal Neurological Institute, McGill University, Montreal, Quebec, H3A 2B4, Canada; Translational Neuroimaging Laboratory, The McGill University Research Centre for Studies in Aging, Douglas Mental Health Institute, Montreal, Quebec, H4H 1R3, Canada; Department of Psychiatry, School of Medicine, University of Pittsburgh, Pittsburgh, PA, 15213, USA; Translational Neuroimaging Laboratory, The McGill University Research Centre for Studies in Aging, Douglas Mental Health Institute, Montreal, Quebec, H4H 1R3, Canada; Department of Neurology and Neurosurgery, McGill University, Montreal, Quebec, H3A 1A1, Canada; Montreal Neurological Institute, McGill University, Montreal, Quebec, H3A 2B4, Canada; Translational Neuroimaging Laboratory, The McGill University Research Centre for Studies in Aging, Douglas Mental Health Institute, Montreal, Quebec, H4H 1R3, Canada; Department of Neurology and Neurosurgery, McGill University, Montreal, Quebec, H3A 1A1, Canada; Montreal Neurological Institute, McGill University, Montreal, Quebec, H3A 2B4, Canada; Translational Neuroimaging Laboratory, The McGill University Research Centre for Studies in Aging, Douglas Mental Health Institute, Montreal, Quebec, H4H 1R3, Canada; Department of Neurology and Neurosurgery, McGill University, Montreal, Quebec, H3A 1A1, Canada; Montreal Neurological Institute, McGill University, Montreal, Quebec, H3A 2B4, Canada; Translational Neuroimaging Laboratory, The McGill University Research Centre for Studies in Aging, Douglas Mental Health Institute, Montreal, Quebec, H4H 1R3, Canada; Department of Neurology and Neurosurgery, McGill University, Montreal, Quebec, H3A 1A1, Canada; Montreal Neurological Institute, McGill University, Montreal, Quebec, H3A 2B4, Canada; Translational Neuroimaging Laboratory, The McGill University Research Centre for Studies in Aging, Douglas Mental Health Institute, Montreal, Quebec, H4H 1R3, Canada; Department of Neurology and Neurosurgery, McGill University, Montreal, Quebec, H3A 1A1, Canada; Montreal Neurological Institute, McGill University, Montreal, Quebec, H3A 2B4, Canada; Translational Neuroimaging Laboratory, The McGill University Research Centre for Studies in Aging, Douglas Mental Health Institute, Montreal, Quebec, H4H 1R3, Canada; Department of Neurology and Neurosurgery, McGill University, Montreal, Quebec, H3A 1A1, Canada; Montreal Neurological Institute, McGill University, Montreal, Quebec, H3A 2B4, Canada; Translational Neuroimaging Laboratory, The McGill University Research Centre for Studies in Aging, Douglas Mental Health Institute, Montreal, Quebec, H4H 1R3, Canada; Translational Neuroimaging Laboratory, The McGill University Research Centre for Studies in Aging, Douglas Mental Health Institute, Montreal, Quebec, H4H 1R3, Canada; Department of Psychiatry, School of Medicine, University of Pittsburgh, Pittsburgh, PA, 15213, USA; Department of Psychiatry, School of Medicine, University of Pittsburgh, Pittsburgh, PA, 15213, USA; Department of Psychiatry, School of Medicine, University of Pittsburgh, Pittsburgh, PA, 15213, USA; Department of Neurology, National Neuroscience Institute, Singapore, 308433, Singapore; Translational Neuroimaging Laboratory, The McGill University Research Centre for Studies in Aging, Douglas Mental Health Institute, Montreal, Quebec, H4H 1R3, Canada; Department of Pharmacology, Graduate Program in Biological Sciences: Pharmacology and Therapeutics, and Biochemistry, Universidade Federal do Rio Grande do Sul, Porto Alegre, 90.035-003, Brazil; Brain Institute of Rio Grande do Sul, PUCRS, Porto Alegre, 90610-000, Brazil; Department of Psychiatry, School of Medicine, University of Pittsburgh, Pittsburgh, PA, 15213, USA; Department of Neurology, School of Medicine, University of Pittsburgh, Pittsburgh, PA, 15213, USA; Translational Neuroimaging Laboratory, The McGill University Research Centre for Studies in Aging, Douglas Mental Health Institute, Montreal, Quebec, H4H 1R3, Canada; Department of Neurology and Neurosurgery, McGill University, Montreal, Quebec, H3A 1A1, Canada; Translational Neuroimaging Laboratory, The McGill University Research Centre for Studies in Aging, Douglas Mental Health Institute, Montreal, Quebec, H4H 1R3, Canada; Department of Neurology and Neurosurgery, McGill University, Montreal, Quebec, H3A 1A1, Canada; Montreal Neurological Institute, McGill University, Montreal, Quebec, H3A 2B4, Canada

**Keywords:** Braak stages, Alzheimer’s disease, PET, neurofibrillary tangles, activities of daily living

## Abstract

The progression of PET-based Braak stages correlates with cognitive deterioration in aging and Alzheimer’s disease. Here, we investigate the association between PET-based Braak stages and functional impairment and assess whether PET-based Braak staging predicts a longitudinal decline in the performance of activities of daily living. In this cohort study, we evaluated cognitively unimpaired individuals and individuals with mild cognitive impairment or Alzheimer’s disease dementia. Participants underwent [^18^F]MK6240 tau-PET, were assigned a PET-based Braak stage at baseline and were followed for a mean (SD) of 1.97 (0.66) years. Functional performance was evaluated with the Functional Activities Questionnaire, Everyday Cognition and functional Clinical Dementia Rating sum of boxes. Multiple linear regressions assessed the association of PET-based Braak stages with baseline functionality and with the longitudinal rate of change in functional scores, adjusting for age, sex and amyloid-β load. We employed voxel-based regression models to investigate the association between functionality and tau-PET signal and assessed the voxel overlap with Braak regions of interest. We included 291 individuals (181 cognitively unimpaired, 56 amyloid-β+ mild cognitive impairment and 54 amyloid-β+ Alzheimer’s disease) aged 70.60 (7.48) years. At baseline, PET-based Braak stages III–IV (*β* = 0.43, *P* = 0.03) and V–VI (*β* = 1.20, *P* < 0.0001) showed associations with poorer Functional Activities Questionnaire scores. Similarly, stages III–IV (*β* = 0.43, *P* = 0.02) and V–VI (*β* = 1.15, *P* < 0.0001) were associated with worse Everyday Cognition scores. Only stages V–VI were associated with higher functional Clinical Dementia Rating sum of boxes (*β* = 1.17, *P* < 0.0001) scores. Increased tau-PET signals in all Braak regions of interest were linked to worse performance in all tools. The voxelwise analysis showed widespread cortical associations between functional impairment and tau-PET and high voxel overlap with Braak regions of interest. Baseline PET-based Braak stages V–VI predicted significant longitudinal functional decline as assessed by the Functional Activities Questionnaire (*β* = 1.69, *P* < 0.0001), the Everyday Cognition (*β* = 1.05, *P* = 0.001) and the functional Clinical Dementia Rating sum of boxes (*β* = 1.29, *P* < 0.0001). Our results suggest that functional impairment increases with the severity of tau accumulation. These findings also indicate that PET-based Braak staging is a good predictor of functional impairment in the Alzheimer’s disease continuum. Finally, our study provides evidence for the clinical significance of the PET-based Braak staging framework.

## Teaser

Macedo *et al*. report that difficulties in the performance of activities of daily living increase with the severity of tau accumulation. Their findings also suggest that Braak staging assigned with PET may be useful to predict functional impairment and to select participants for clinical trials targeting Alzheimer’s disease.

## Graphical Abstract

**Graphical Abstract:**
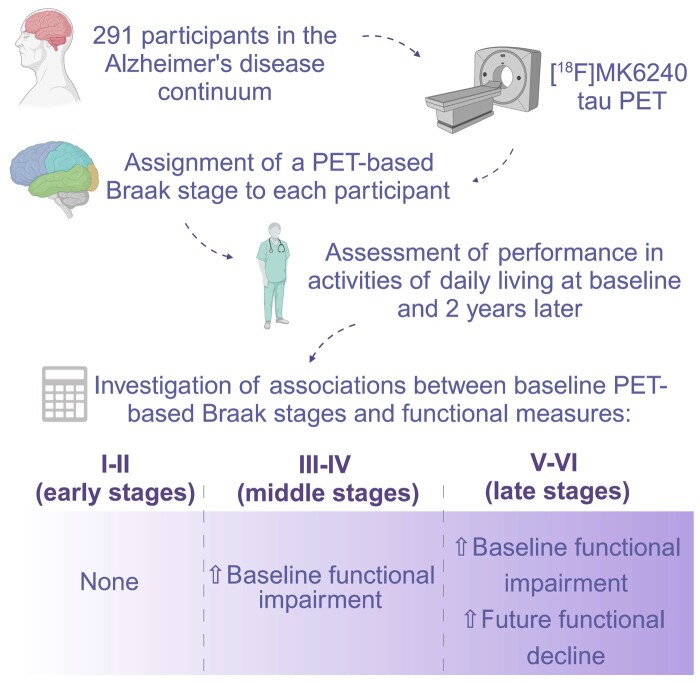

## Introduction

Alzheimer’s disease is characterized by abnormal accumulation of amyloid-β (Aβ) and neurofibrillary tangles (NFTs) in the brain, which begin decades before symptom onset.^1^ Based on the NFT accumulation pattern, Braak and Braak^2^ proposed a histopathological classification for Alzheimer’s disease comprising six successive stages ranging from transentorhinal cortex involvement (stage I) to degeneration of primary sensory cortices (stage VI). The Braak staging scheme accurately indicates the severity of NFT spreading in the brain, having been included in the neuropathological diagnostic criteria for Alzheimer’s disease.^3^ However, employing the Braak staging scheme in living humans is impossible because it relies on post-mortem observations.

PET imaging of tau NFT is one of the biomarkers representing the ‘T’ category of the AT(N) system proposed by the National Institute on Aging and Alzheimer’s Association (NIA-AA) research criteria for Alzheimer’s disease.^4^ Given that tau-PET measures the regional NFT deposition in the brain, it allows for the stratification of individuals in the Alzheimer’s disease continuum based on *in vivo* tau Braak staging.^5-10^ While the progression of PET-based Braak stages was shown to correlate with clinical deterioration in the Alzheimer’s disease continuum, most studies have focused on cognitive and global disease severity measures and not on the level of independence in activities of daily living (ADL).^7-11^

An impaired performance on ADL is one of the required NIA-AA clinical diagnostic criteria for probable Alzheimer’s disease dementia.^12^ Even though mildly impaired complex instrumental ADL (IADL) may also be observed in mild cognitive impairment (MCI) patients,^13^ functional decline in ADL is a critical clinical feature to stratify individuals in the Alzheimer’s disease continuum and assess disease severity. Based on their complexity, ADL may be divided into three categories: (i) basic ADL (BADL; self-care tasks such as bathing and feeding); (ii) IADL (activities to maintain an independent household such as doing housework, taking medications and financial activities); and (iii) advanced ADL (AADL; related to the performance of societal, community and family roles).^14^ Impairments in more complex tasks (IADL and AADL) tend to precede those in BADL with the progression of cognitive deterioration.^15-17^

The relationship between functional autonomy in ADL and PET-based Braak staging is yet to be clarified. In the present study, we aim to investigate the association between PET-based Braak stages and functional impairment as assessed by tools used in clinical practice to diagnose dementia. We also assessed whether PET-based Braak staging predicts a longitudinal decline in the performance of ADL. Based on the assumption that functional decline is due to cognitive impairment, as stated in the dementia diagnostic criteria,^12^ we hypothesize that functional impairment will be associated with middle to late PET-based Braak stages (III–VI). As a secondary aim, we investigated the impact of these associations to the population recruitment of randomized controlled trials (RCTs) using measures of functionality as outcomes.

## Materials and methods

### Participants

In this prospective longitudinal study, we evaluated cognitively unimpaired (CU) individuals and individuals diagnosed with either MCI or Alzheimer’s disease dementia from the Translational Biomarkers in Aging and Dementia (TRIAD) cohort.^18^ CU individuals were recruited from the community, while individuals with MCI or Alzheimer’s disease dementia were recruited from the community or the McGill Research Centre of Studies on Aging outpatient memory clinic, in Montreal, Quebec, Canada. Recruitment took place from July 2016 to December 2021 through referrals, advertisements and word of mouth. CU participants were those with no objective cognitive impairment. Following the assessment of a multidisciplinary team composed of neurologists, neuropsychologists and nurses, participants with MCI and participants with Alzheimer’s disease dementia met, respectively, the NIA-AA criteria for MCI due to Alzheimer’s disease^19^ and for probable Alzheimer’s disease dementia.^12^ Of note, the Alzheimer’s disease dementia group included individuals with both the amnestic and non-amnestic variants—behavioural/dysexecutive Alzheimer’s disease,^20^ logopenic primary progressive aphasia^21^ and posterior cortical atrophy.^22^ The Aβ status of participants was assessed at baseline using [^18^F]AZD4694 Aβ PET, and, similar to previous research,^23^ only Aβ+ MCI and Aβ+ Alzheimer’s disease individuals were included since our aim was to assess functional performance in the aging and Alzheimer’s disease continuum. Participants also underwent [^18^F]MK6240 tau-PET and brain MRI at baseline. Potential participants were excluded if they presented visual and auditory impairments that hampered neuropsychological evaluation, inability to speak French or English, recent traumatic brain injury or major surgery, MRI/PET contraindications or inadequately treated neurological, psychiatric or systemic disorders. In case a reliable informant (e.g. a family member or a close friend) was unavailable, potential participants were also excluded. A subset of participants returned for a follow-up visit, in which another clinical assessment was performed. This study received approval from the Montreal Neurological Institute PET working committee and the Douglas Mental Health University Institute Research Ethics Board (IUSMD 16-60). Participants signed informed consent forms after being explained about all research procedures.

### Neuroimaging acquisition and processing

A 3 T Siemens Magnetom scanner using a standard head coil was used to acquire structural brain MRI, while a brain-dedicated Siemens high-resolution research tomograph was employed to acquire [^18^F]AZD4694 and [^18^F]MK6240 PET scans. The injected radiation activity per participant per PET scan ranged from 5 to 7 mCi. Tau-PET images were obtained after 90–110 min of the [^18^F]MK6240 intravenous bolus injection and reconstructed employing a sequential subset expectation–maximization algorithm on a 4D volume with four frames (4 × 300 s), as previously reported.^24^ The acquisition of Aβ PET images was done after 40–70 min of the [^18^F]AZD4694 intravenous injection; reconstruction was performed with a sequential subset expectation–maximization algorithm on a 4D volume with three frames (3 × 600 s).^25^ In order to correct the attenuation, a 6-min transmission scan with a rotating 137Cs point source was performed following each PET acquisition. Corrections for motion, dead time, decay and random and scattered coincidences were performed. Subsequently, PET images were linearly registered to T_1_-weighted MRI image space and then linearly and nonlinearly registered to the Montreal Neurological Institute reference space. Furthermore, PET images were smoothed spatially to provide a full width of 8 mm at a half-maximum resolution. Meninges were further stripped in native space from [^18^F]MK6240 PET images before transformations and blurring to prevent meningeal spill-over into adjacent brain areas.^7^ The standardized uptake value ratio (SUVR) in [^18^F]MK6240 PET was determined using the cerebellum crus I grey matter as the reference region.^9,26^ To calculate [^18^F]AZD4694 SUVRs, the whole cerebellum grey matter was used as the reference region, and the following regions were included in the global [^18^F]AZD4694 SUVR composite: precuneus, prefrontal, orbitofrontal, parietal, temporal and cingulate cortices.^25^ The threshold to consider [^18^F]AZD4694 SUVR as positive was established as an SUVR > 1.55, as previously described.^25^

### PET-based Braak staging methods

PET-based Braak stages were defined as reported elsewhere.^7^ The Braak regions of interest (ROIs) were defined using the Desikan–Killiany–Tourville atlas.^27^ The transentorhinal cortex underwent segmentation as previously described.^6,7,9^

Each individual was assigned a PET-based Braak stage based on the latest stage in which tau-PET was found to be abnormal. The thresholds for this abnormality in Braak ROIs were calculated as 2.5 SD higher than the mean SUVR of CU aged <26 years, as reported elsewhere.^6,7^ Aiming to increase the sample size in each Braak staging group, participants were grouped according to the simplified Braak staging system into four different PET-based Braak staging groups: 0, I–II, III–IV and V–VI.^2^

### Clinical and neuropsychological assessments

All participants underwent a detailed clinical and neuropsychological evaluation. Functional impairment was evaluated with the Functional Activities Questionnaire (FAQ), the Everyday Cognition (ECog) and the Clinical Dementia Rating sum of boxes (CDR-SB) functional domains.

The FAQ is a 10-item scale in which an informant rates the participant’s ability on 10 IADLs.^28^ Each item is scored from 0 (normal) to 3 (dependent), with the total score ranging from 0 to 30 and higher scores reflecting poorer functioning.^28^

The ECog measures subtle and mild functional changes in older adults that are relevant to certain cognitive domains: memory, language, visual–spatial and perceptual abilities, and executive functioning (planning, organization and divided attention).^29^ In our study, this questionnaire was answered by an informant who rated the current ability of the participant to accomplish specific tasks as compared to their performance 10 years ago. The responses to the 39 questions range from 1 (better or no change) to 4 (consistently much worse), with the final score being the average of all answers. A higher average score means greater impairment.^29^

The CDR-SB assesses the impact of cognitive deficits on the performance of everyday activities and was developed as a tool to stage dementia severity.^30^ It consists of a semi-structured interview with the participant and an informant, who is asked to describe the participant’s degree of impairment. The scale includes three cognitive (‘memory’, ‘orientation’, and ‘judgment and problem solving’) and three functional domains (‘community affairs’, ‘home and hobbies’, and ‘personal care’), whose scores vary from 0 (healthy) to 3 (severe dementia).^30^ Combined, the scores of the functional domains result in the ‘functional CDR-SB’ (CDR-SB-F), with scores ranging from 0 to 9.

We selected these tools, which are commonly used in research and clinical practice, to compare their relationship with PET-based Braak staging. Considering that the FAQ and the ECog encompass questions regarding more complex ADL, we expected associations with earlier PET-based Braak stages than the CDR-SB-F, which also evaluates the performance in BADL.

### Statistical analysis

Statistical analyses were conducted on R software version 3.5.3. Demographic data were compared using the Kruskal–Wallis for continuous variables [age, years of education, Mini-Mental State Examination (MMSE) and SUVRs] and *χ*^2^ test for categorical variables (sex and APOE ε4 status). In addition, we compared the functional scores of participants at different PET-based Braak stages using one-way analysis of variance (ANOVA) and performed *post hoc* analyses with Tukey’s honest significance test. Multiple linear regression (MLR) was used to assess the association of PET-based Braak stages and Braak ROI SUVR with functional measures at baseline. We also employed voxel-based regression models to investigate the association between functional scores and tau-PET signal at baseline, adjusting for age, sex and total Aꞵ PET load. The voxel-based analyses were conducted using VoxelStats,^31^ a MATLAB package, and corrected for multiple comparisons with the random field theory method employing a threshold of *P* < 0.001. Subsequently, we calculated the voxel overlap between the t-maps generated from the voxelwise analysis and the masks for each Braak ROI. Furthermore, we employed MLR to investigate whether baseline PET-based Braak stages predicted the annual change in functional scores, as most participants had a single follow-up timepoint. Briefly, the annual change was calculated as the difference between the follow-up and baseline scores divided by the time (in years) between the visits. For the annual change, we considered data points below 2.5 SD than the mean of the whole sample to be outliers. These outliers were excluded from any analyses due to the lack of biological and clinical plausibility. All MLR analyses were adjusted for age, sex and neocortical [^18^F]AZD4694 Aꞵ PET SUVR. We also ran exploratory models adjusted for clinical diagnosis to understand its contribution to the relationship between Braak staging and ADL performance. All continuous variables added to the MLR models were Z-scored based on the whole sample. Statistical significance was considered if *P* < 0.05. Finally, we calculated sample size required in a hypothetical RCT to observe 25% reduction in functional decline in the treatment group, with a power of 80%, using a well-validated formula.^32^

## Results

A PET-based Braak stage was assigned to 291 individuals (112 at stage 0, 78 at I/II, 35 at III/IV and 66 at V/VI), who had a mean age of 70.60 (7.48) and were 62.8% (*n* = 183) females (Table 1). In total, 181 participants were classified as CU, 56 as Aβ+ MCI and 54 as Aβ+ Alzheimer’s disease. Significant differences between groups were observed for age, clinical diagnosis, MMSE scores, APOE ε4 status and [^18^F]AZD4694 PET SUVR.

**Table 1:** Demographic, clinical and biomarker characteristics of the study cohort at baseline

	Braak 0 (*N* = 73)	Braak I-II (*N* = 58)	Braak III-IV (*N* = 21)	Braak V-VI (*N* = 36)	*P*-value
Age (years), mean (SD)	69.93 (7.6)	72.23 (6.8)	72.54 (5.0)	68.77 (8.6)	**0.01**
Female, *n* (%)	68 (60.7%)	55 (70.5%)	22 (62.9%)	38 (57.6%)	0.40
Years of education, mean (SD)	15.64 (3.7)	15.22 (3.8)	15.94 (3.5)	14.65 (3.9)	0.37
Clinical diagnosis, *n* (%)	108 CU (96.4%); 4 MCI (3.6%)	56 CU (71.8%); 19 MCI (24.4%); 3 Alzheimer’s disease (3.8%)	17 CU (48.6%); 11 MCI (31.4%); 7 Alzheimer’s disease (20.0%)	22 MCI (33.3%); 44 Alzheimer’s disease (66.7%)	**<0.001**
MMSE, mean (SD)^a^	29.07 (1.1)	28.76 (1.7)	28.03 (3.1)	22.00 (6.2)	**<0.001**
APOE ε4 carriers, *n* (%)^b^	24 (21.4%)	29 (37.2%)	18 (51.4%)	35 (53.0%)	**<0.001**
Neocortical [^18^F]AZD4694 SUVR, mean (SD)^c^	1.38 (0.3)	1.75 (0.5)	2.01 (0.6)	2.64 (0.5)	**<0.001**

*P*-values were calculated using the Kruskal–Wallis test for age, years of education, MMSE and SUVRs. *χ*^2^ test was performed for sex proportion and APOE ε4 status comparison. Statistical significance was considered if *P* < 0.05 (results in bold). CU, cognitively unimpaired; MCI, mild cognitive impairment; MMSE, Mini-Mental State Examination; ROI, region of interest; SD, standard deviation; SUVR, standardized uptake value ratio. a Two missing data. b Twenty missing data. c Three missing data.

A total of 188 participants returned for a follow-up visit after a mean (SD) of 1.97 (0.66) years. The minimum and maximum follow-up times were 0.8 and 3.6 years, respectively. No significant difference was observed in the groups’ follow-up time. A complete description of their demographic, clinical and biomarkers characteristics is displayed in Supplementary Table 1.

**Figure 1:**
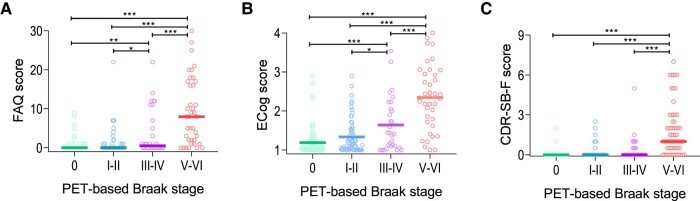
**Progression of functional decline across PET-based Braak stages.** The graphs depict the median baseline scores obtained by participants at different PET-based Braak stages in the FAQ (**A**), ECog (**B**), and CDR-SB-F (**C**). Comparisons were performed with one-way ANOVA and *post hoc* analyses with Tukey’s honest significance test. CDR-SB-F, functional Clinical Dementia Rating sum of boxes; ECog: Everyday Cognition; FAQ: Functional Activities Questionnaire. *P < 0.05. **P < 0.01. ***P < 0.001.

**Figure 2:**
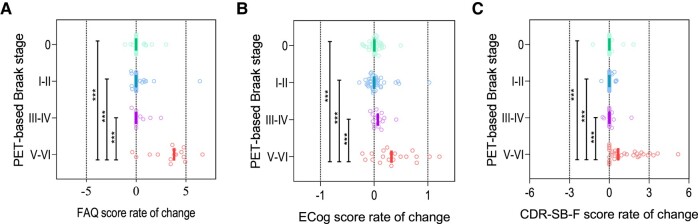
**Annual change in the functional scores according to baseline PET-based Braak stage.** The graphs show the median annual change in the functional scores (**A**, FAQ; **B**, ECog; and **C**, CDR-SB-F) per PET-based Braak stage. Comparisons were performed with one-way ANOVA and *post hoc* analyses with Tukey’s honest significance test. CDR-SB-F, functional Clinical Dementia Rating sum of boxes; ECog, Everyday Cognition; FAQ: Functional Activities Questionnaire. ***<0.001.

For all tools, ANOVA analyses indicated significant differences in the scores of participants at different Braak stages, both regarding baseline measures and rates of change (*P* < 0.0001 for all comparisons). Significantly lower baseline functional scores, as compared to stage 0, started to be observed at stages III–IV for the FAQ and the ECog and at stages V–VI for the CDR-SB-F (Fig. 1A–C). In turn, a significantly higher annual decline in functionality was seen at stages V–VI for all tools (Fig. 2A–C). Importantly, three outliers for the annual rate of change variables were identified and excluded.

In the cross-sectional analysis (Table 2), PET-based Braak stages III–IV [*β* = 0.43, 95% confidence interval (CI) 0.05–0.81, *P* = 0.03] and V–VI (*β* = 1.20, 95% CI 0.75–1.65, *P* < 0.0001) were associated with worse FAQ scores. Similarly, a significant association was also found between stages III–IV (*β* = 0.43, 95% CI 0.07–0.79, *P* = 0.02) and V–VI (*β* = 1.15, 95% CI 0.73–1.58, *P* < 0.0001) and a poorer performance in the ECog. Only stages V–VI, however, were associated with higher scores in the CDR-SB-F (*β* = 1.17, 95% CI 0.80–1.54, *P* < 0.0001).

**Table 2:** Regression coefficients of the cross-sectional association between PET-based Braak stages and functional scores at baseline

	FAQ			ECog			CDR-SB-F		
	Beta (95% CI)	*T*-value	*P*-value	Beta (95% CI)	*T*-value	*P*-value	Beta (95% CI)	*T*-value	*P*-value
PET-based Braak stage I–II	0.02 (−0.27 to 0.31)	0.15	0.88	0.08 (−0.19 to 0.36)	0.60	0.55	0.05 (−0.20 to 0.30)	0.39	0.70
PET-based Braak stage III–IV	0.43 (0.05–0.81)	2.24	**0.03**	0.43 (0.07–0.79)	2.37	**0.02**	0.15 (−0.18 to 0.49)	0.89	0.38
PET-based Braak stage V–VI	1.20 (0.75–1.65)	5.24	**<0.0001**	1.15 (0.73–1.58)	5.36	**<0.0001**	1.17 (0.80–1.54)	6.18	**<0.0001**
Age (years)	−0.12 (−0.25 to 0.003)	−1.93	0.06	−0.13 (−0.25 to −0.01)	−2.19	**0.03**	−0.09 (−0.19 to 0.008)	−1.80	0.07
Sex (male)	0.08 (−0.14 to 0.31)	0.73	0.47	0.13 (−0.09 to 0.34)	1.18	0.24	0.07 (−0.13 to 0.27)	0.71	0.48
Neocortical [^18^F]AZD4694 SUVR	0.18 (0.007–0.36)	2.05	**0.04**	0.28 (0.12–0.45)	3.36	**<0.001**	0.16 (0.02–0.30)	2.26	**0.02**

Adjusted *R*^2^: 0.33, *F*-stat: 19.39 (FAQ); adjusted *R*^2^: 0.41, *F*-stat: 26.01 (ECog); adjusted *R*^2^: 0.35, *F*-stat: 26.83 (CDR-SB-F). Statistical significance was considered if *P* < 0.05 (results in bold). CDR-SB-F, functional Clinical Dementia Rating sum of boxes; ECog, Everyday Cognition; FAQ, Functional Activities Questionnaire; SUVR, standardized uptake value ratio.

**Figure 3:**
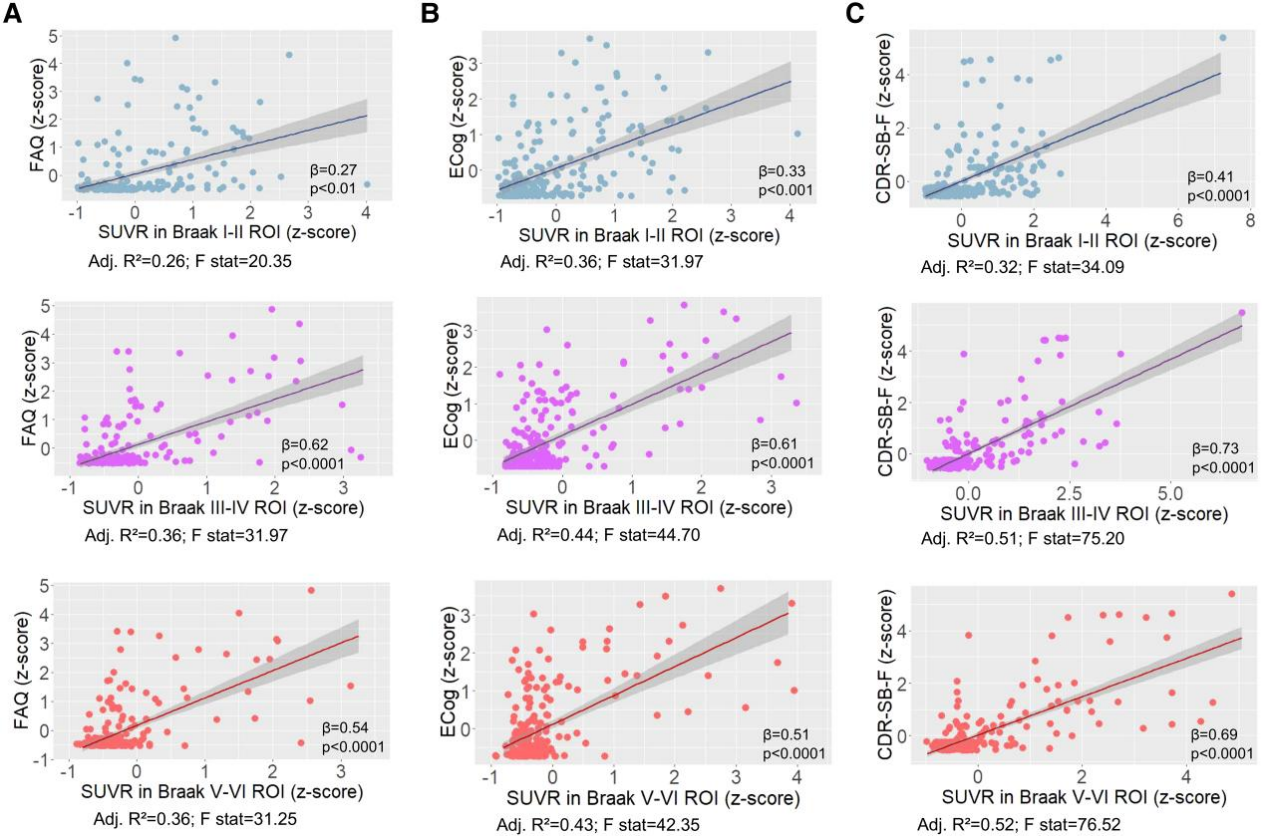
**Association between the SUVR in Braak ROIs and functional scores at baseline.** The graphs depict the association between tau-PET SUVR in Braak regions and functional performance as assessed by the FAQ (**A**), the ECog (**B**), and the CDR-SB-F (**C**). Depicted *β*-coefficients and *P*-values were obtained in linear regression models adjusted for age, sex and neocortical [^18^F]AZD4694 Aꞵ PET SUVR. CDR-SB-F, functional Clinical Dementia Rating sum of boxes; ECog, Everyday Cognition; FAQ, Functional Activities Questionnaire; ROI, region of interest; SUVR, standardized uptake value ratio.

Increased SUVR in Braak I–II (*β* = 0.27, 95% CI 0.09–0.45, *P* = 0.004), III–IV (*β* = 0.62, 95% CI 0.43–0.80, *P* < 0.0001) and V–VI (*β* = 0.54, 95% CI 0.38–0.71, *P* < 0.0001) ROIs was significantly associated with worse performance in the FAQ (Fig. 3A). Significant associations were also observed between higher SUVR in Braak I–II (*β* = 0.33, 95% CI 0.80–1.54, *P* < 0.001), III–IV (*β* = 0.61, 95% CI 0.44–0.78, *P* < 0.0001) and V–VI (*β* = 0.51, 95% CI 0.36–0.67, *P* < 0.0001) and poorer ECog scores (Fig. 3B). Higher tau-PET SUVR in all Braak ROIs (I–II: *β* = 0.41, 95% CI 0.26–0.57, *P* < 0.0001; III–IV: *β* = 0.73, 95% CI 0.62–0.85, *P* < 0.0001; and V–VI: *β* = 0.69, 95% CI 0.58–0.80, *P* < 0.0001) was also associated with an increased impairment as assessed by the CDR-SB-F (Fig. 3C).

**Figure 4:**
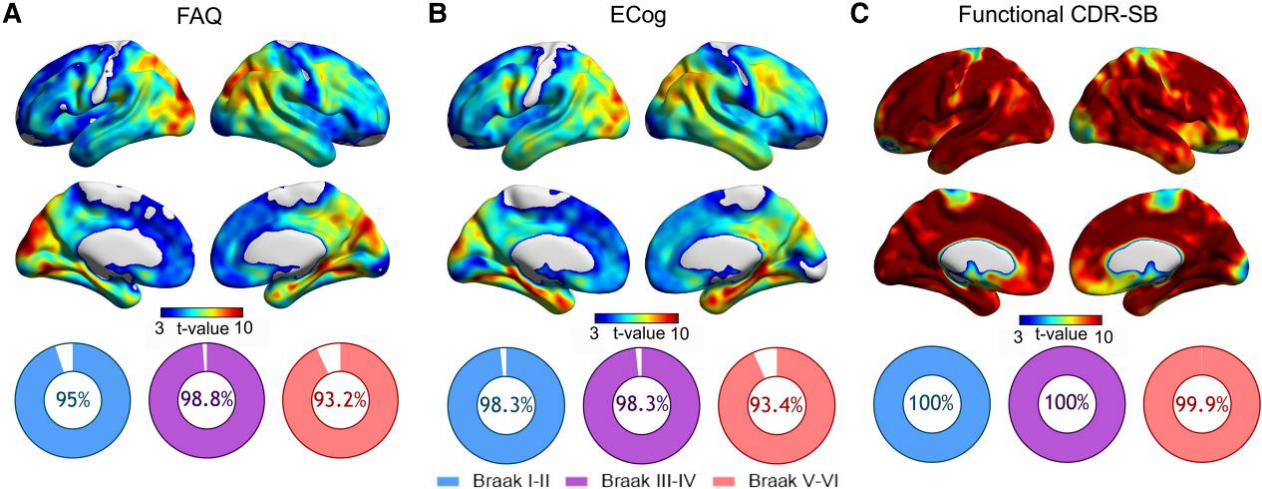
**Tau-PET correlates of functional impairment and their overlap with Braak ROIs.** The images show the *T*-values resulting from voxel-based linear regressions assessing the associations between [^18^F]MK6240 SUVR and performance at the FAQ (**A**), ECog (**B**), and CDR-SB-F (**C**). Adjustment was performed for age, sex and total [^18^F]AZD4694 Aꞵ PET burden. The pie graphs represent the proportion of overlap between the generated t-maps and the masks representing each Braak ROI. CDR-SB, Clinical Dementia Rating sum of boxes; ECog, Everyday Cognition; FAQ, Functional Activities Questionnaire; ROI, region of interest; SUVR, standardized uptake value ratio.

The voxelwise analysis showed the most notable associations between FAQ scores and tau-PET SUVR in the left precentral and lateral occipital gyri, right isthmus cingulate and bilaterally in the supramarginal, superior temporal, superior parietal, inferior parietal, lingual and fusiform gyri, as well as in the precuneus, cuneus and hippocampi (Fig. 4A). In turn, the highest *T*-values for the ECog were seen in the cuneus, precuneus, inferior parietal and isthmus of the cingulate, in the right hemisphere; in the precentral, superior parietal and lateral occipital gyri, in the left hemisphere; and in the entorhinal, hippocampal, parahippocampal, fusiform, lingual, superior temporal and inferior temporal cortices, bilaterally (Fig. 4C). For the CDR-SB-F, widespread strong clusters were observed across the whole cerebral cortex (Fig. 4C).

For all functional outcomes, the voxelwise analysis showed a high topographic overlap between the significant voxels and the masks for Braak ROIs (Fig. 4A–C). The FAQ t-map had an overlap with the Braak I–II, III–IV and V–VI masks of 95%, 98.8% and 93.2%, respectively. The overlap of the ECog t-map was of 98.3% with Braak I–II, 98.3% with III–IV and 93.4% with V–VI. Finally, the CDR-SB-F t-map displayed the highest overlaps: 100% with Braak I–II and III–IV, and 99.9% with Braak V–VI ROIs.

In the longitudinal analysis (Table 3), baseline PET-based Braak stages V–VI predicted a significantly higher rate of increase in the FAQ (*β* = 1.69, 95% CI 1.05–2.32, *P* < 0.0001), the ECog (*β* = 1.05, 95% CI 0.42–1.69, *P* = 0.001) and the CDR-SB-F (*β* = 1.29, 95% CI 0.79–1.78, *P* < 0.0001). The same significant associations were found previously to the elimination of the three outliers (Supplementary Table 2).

**Table 3:** Regression coefficients of the association between baseline PET-based Braak stages and the annual change in the functional scores

	FAQ			ECog			CDR-SB-F		
	Beta (95% CI)	*T*-value	*P*-value	Beta (95% CI)	*T*-value	*P*-value	Beta (95% CI)	*T*-value	*P*-value
PET-based Braak stage I–II	0.01 (−0.34 to 0.36)	0.06	0.95	0.003 (−0.39 to 0.39)	0.01	0.99	−0.04 (−0.34 to 0.26)	−0.26	0.79
PET-based Braak stage III–IV	0.02 (−0.47 to 0.51)	0.08	0.93	0.02 (−0.52 to 0.57)	0.08	0.93	−0.03 (−0.45 to 0.40)	−0.12	0.91
PET-based Braak stage V–VI	1.69 (1.05–2.32)	5.29	**<0**.**0001**	1.05 (0.42–1.69)	3.29	**0**.**001**	1.29 (0.79–1.78)	5.17	**<0**.**0001**
Age (years)	−0.05 (−0.25 to 0.15)	−0.53	0.59	0.07 (−0.15 to 0.28)	0.59	0.55	−0.14 (−0.29 to 0.01)	−1.80	0.07
Sex (male)	0.02 (−0.27 to 0.32)	0.17	0.87	0.15 (−0.18 to 0.49)	0.91	0.36	0.02 (−0.23 to 0.26)	0.12	0.90
Neocortical [^18^F]AZD4694 SUVR	0.21 (−0.04 to 0.45)	1.70	0.09	0.25 (0.006–0.50)	2.03	**0**.**04**	0.15 (−0.05 to 0.34)	1.50	0.14

Adjusted *R*^2^: 0.50, *F*-stat: 17.61 (FAQ); adjusted *R*^2^: 0.26, *F*-stat: 8.36 (ECog); adjusted *R*^2^: 0.36, *F*-stat: 18.15 (CDR-SB-F). Statistical significance was considered if *P* < 0.05 (results in bold). CDR-SB-F, functional Clinical Dementia Rating sum of boxes; ECog, Everyday Cognition; FAQ, Functional Activities Questionnaire; SUVR, standardized uptake value ratio.

In a hypothetical RCT, for a 25% reduction in functional decline to be observed, the required sample size per study arm would range from 78 to 288 individuals at Braak stages V–VI, depending on the questionnaire used (Table 4). In case individuals at earlier stages were to be recruited, for a similar performance of the drug, the sample size would have to be 2.13–50.65 times bigger.

**Table 4:** The minimum sample size required per RCT arm to observe a considerable reduction in functional decline

	FAQ	ECog	CDR-SB-F
Braak I–II	3951	6925	4020
Braak III–IV	1170	565	8204
Braak V–VI	78	265	288

The table contains the calculation of the minimum sample required for a 25% slowing in functional decline to be observed, considering the baseline PET-based Braak stage of participants. CDR-SB-F, functional Clinical Dementia Rating sum of boxes; ECog, Everyday Cognition; FAQ, Functional Activities Questionnaire; RCT, randomized controlled trial.

### Exploratory analysis

We conducted exploratory analyses with models adjusting for clinical diagnosis. Statistical significance was lost for the associations between baseline functional scores and baseline PET-based Braak stages (Supplementary Table 3). On the other hand, statistical significance remained for the relationship between Braak III–IV SUVR and ECog scores (*β* = 0.18, 95% CI 0.01–0.35, *P* = 0.04) as well as between CDR-SB-F scores and SUVR in Braak I–II (*β* = 0.24, 95% CI 0.11–0.37, *P* < 0.001), III–IV (*β* = 0.45, 95% CI 0.32–0.58, *P* < 0.0001) and V–VI (*β* = 0.43, 95% CI 0.31–0.55, *P* < 0.0001). Baseline Braak V–VI remained associated with the annual rate of change in the FAQ (*β* = 1.28, 95% CI 0.63–1.93, *P* < 0.001) and in the CDR-SB-F (*β* = 0.65, 95% CI 0.13–1.17, *P* = 0.01) but not in the ECog (Supplementary Table 4).

## Discussion

In the present study, we applied *in vivo* PET-based Braak staging to evaluate the association between cerebral NFT distribution and functional impairment. Our findings demonstrate that functional decline, a core criterion of dementia diagnosis,^12^ is associated with middle to late PET-based Braak stages. Additionally, we show that PET-based Braak staging can predict a longitudinal decline in the performance of ADL. Taken together, these results are in agreement with previous data suggesting that the progression of PET-based Braak stages has a good correspondence with clinical deterioration in the Alzheimer’s disease continuum.^7-11^ These findings also provide *in vivo* evidence that functional impairment increases with the severity of NFT accumulation.

Results of the FAQ are consistent with our hypothesis and with findings showing that IADL deficits are observed either in prodromal (MCI) or, most commonly, early dementia because they repose upon more complex cognitive functions, which are affected earlier in the disease.^14^ Similarly, the association of PET-based Braak stages III–IV and V–VI with functional impairment in the ECog might be explained by the focus of this scale on subtle cognitive changes, especially memory, which have been known to occur earlier in Alzheimer’s disease pathogenesis.^33^ Even though the CDR-SB-F includes domains evaluating tasks of higher complexity (i.e. ‘community affairs’ and ‘home and hobbies’), no association with middle PET-based Braak stages was observed. This is possibly explained by the fact that the CDR-SB-F includes a domain assessing BADL (i.e. ‘personal care’). Nonetheless, we found that decreased functionality, regardless of the tool used, is associated with higher tau-PET signal in all Braak ROIs. These findings were corroborated by voxelwise analyses showing several significant clusters in Braak-related areas.

Moreover, participants at Braak V/VI, but not at lower stages, had significantly higher rates of functional decline than controls. This indicates not only a potential prognostic value of PET-based Braak staging but also its possible utility when recruiting patients for clinical trials. Indeed, tau-PET staging has been employed to select patients for RCTs of disease-modifying therapies for Alzheimer’s disease. For instance, the phase II and III donanemab RCTs showed higher efficacy among Aβ+ individuals with lower tau burden as assessed by tau-PET.^34,35^ Here, we indicate that functionality might be a useful outcome in trials targeting participants at advanced but not early stages of tau accumulation. The inclusion of participants at lower Braak stages requires much larger sample sizes for considerable slowing in functional decline to be observed, leading to increased costs and logistic challenges.

The literature regarding the relationship between functional impairment and PET-based Braak stages is still scarce. A study using tau-PET [^18^F]AV1451 found an association between poorer CDR-SB scores and higher SUVR in ROIs corresponding to Braak stages I-IV in the MCI group.^36^ Among Alzheimer’s disease individuals, impairment on the CDR-SB correlated with higher SUVR in regions belonging to Braak stages III-VI.^36^ Nonetheless, no specific analyses were performed with the functional domains of the CDR-SB.^36^ These results, however, reinforce the correspondence between CDR-SB scores and PET-based Braak stages in the Alzheimer’s disease continuum. Our results are also in line with neuropathological findings suggesting that higher Braak stages correlate with worse antemortem performances in two functional measures: the FAQ^37^ and the Functional Assessment Staging.^38^ Furthermore, in a small study including 52 participants, Qiu *et al*.^39^ investigated the association of Braak staging with antemortem ECog average score and subscores. Diverging from our findings, this study found no significant correlation, which may be explained by the underpowered sample.^39^

Previous studies investigated tau-PET correlates of functional impairment and returned conflicting results. In individuals across the Alzheimer’s disease continuum, higher FAQ scores showed correlations with increased signal in the precuneus and in a global tau ROI, but only the latter survived correction for Aβ load.^40^ Higher FAQ scores also correlated with tau accumulation in the entorhinal and inferior temporal cortices in another sample of CU and CI participants, but no adjustment for Aβ load was performed.^41^ Moreover, informant-rated but not self-reported poorer performance in ADL was linked to signal in the entorhinal and inferior temporal cortices among CU participants, even though statistical significance was lost after correction for Aβ-PET burden.^42^ In a sample of CI individuals, functional impairment demonstrated associations with greater signal in several ROIs (anterior cingulate, bilateral dorsolateral prefrontal, entorhinal cortex, inferior temporal, lateral parietal, medial orbitofrontal and medial occipital regions), which did not survive adjustment for age, sex and global cognition.^43^

Discordance is also seen in longitudinal data. In 74 Aβ+ CI participants at early symptomatic stages, no associations were observed between [^18^F]PI2620 signal and baseline or longitudinal FAQ scores, in models with stepwise adjustment for age, sex and Aβ-PET.^44^ Meanwhile, baseline [^18^F]AV1451 signal in different ROIs (bilateral entorhinal cortex, inferior temporal, precuneus, posterior cingulate, supramarginal and the dorsolateral prefrontal) predicted increase in FAQ scores in a sample of 334 CU and 247 CI participants, even with adjustments for age, gender, the interaction of baseline age with time, cognitive measures and Aβ-PET.^45^ Here, we add to these findings by showing that a tau-PET staging approach might be useful to predict future functional decline in the Alzheimer’s disease continuum.

Unlike functional assessments, multiple studies investigated the relationship between cognitive performance and PET-based Braak stages.^10,11^ Notably, studies using [^18^F]MK6240 have shown positive correlations between PET-based Braak stages and cognitive impairment in the Alzheimer’s disease continuum. In a cross-sectional study using [^18^F]MK6240 PET, Pascoal *et al*.^7^ demonstrated that the ligand signal in Braak regions was negatively associated with MMSE scores, especially in later stages (V–VI). In an ordinal logistic regression analysis, [^18^F]MK6240 six-stage Braak model was highly correlated with CDR and MMSE scores.^7^ Similarly, another cross-sectional study using [^18^F]MK6240 PET found that higher ligand binding in all Braak stages correlated with poorer MMSE scores.^8^ In a study including 324 participants, Therriault *et al*.^9^ also suggested that [^18^F]MK6240 PET-based Braak stage is associated with cognitive impairment and dementia severity. Specifically, early PET-based Braak stages correlated with isolated memory deficits, while late stages correlated with dementia severity as measured by the CDR.^9^ Overall, cognitive symptoms appeared around PET-based Braak stages II–IV and had their progression associated with the advance of the stages.^9^ Our results regarding functional decline complement these findings and provide further evidence of the good clinical correspondence of the PET-based Braak staging framework with the Alzheimer’s disease continuum.

Interestingly, our findings demonstrated large variability in the functional scores of participants in the same PET-based Braak stage. Although cognitive decline is related to ADL impairment^46^ and associated with the progression of PET-based Braak stages,^7-11^ cognitive reserve may explain the variability of functional scores at the same Braak stage.^47^ Furthermore, even though individuals with ADL impairment present worse overall cognition,^48^ the ADL functional status in individuals with dementia varies significantly relative to the MMSE score.^49^ It should be noted that, besides cognitive impairment, other factors may also lead to functional decline in older adults, such as neuropsychiatric symptoms^50^ and other comorbidities.^51^

Some limitations of our study must be pointed out. Firstly, our population did not include patients with a CDR > 2 or living in nursing homes, whose functional impairment is expected to be greater, adding a selection bias to be considered. Moreover, our sample is mostly composed of White participants, limiting the generalization of our findings to other populations. Another external validity issue is the instruments we used to assess functionality were designed to target symptomatic populations rather than patients at preclinical stages. The absence of neuropathological confirmation at autopsy, the gold standard method to stage individuals according to the Braak framework, is another caveat. Even though the correspondence between [^18^F]MK6240 PET-based and autopsy-assigned Braak staging was shown by a recent study,^6^ there is still the need for studies including larger and more diverse samples to strengthen our confidence in these findings. In addition, case-to-autopsy studies must be performed to confirm that [^18^F]MK6240 PET can detect NFT in early Braak regions. Nonetheless, [^18^F]MK6240 uptake pattern was shown to reliably recapitulate late Braak stage deposition.^7^ PET imaging is also known for its limited spatial resolution, which makes it challenging to detect tau pathology in small areas of the medial temporal lobe. The use of functionality measures based on information obtained from a third party should also be highlighted, since it may lead to informant bias, which we tried to reduce by choosing reliable informants. Nonetheless, using informant-based questionnaires in cognitively impaired individuals is important as they may lack awareness regarding their condition.

In conclusion, our study demonstrates that functional impairment is associated with middle and late PET-based Braak stages and increases with the severity of NFT pathological changes. Moreover, we provide evidence that the PET-based Braak staging may be a good prognostic tool in patients in the Alzheimer’s disease continuum regarding their independence to perform ADL. Our results suggest a good clinical correspondence between the Alzheimer’s disease continuum and the PET-based Braak staging framework, which could be useful to define recruitment strategies for trials of Alzheimer’s disease disease-modifying treatments. Future research should assess the discriminatory and psychometric properties of each scale regarding PET-based Braak stages and compare the progression of other functional outcomes across PET-based Braak stages, including performance-based and self-reported measures.

## Supplementary Material

fcae043_Supplementary_Data

## Data Availability

All requests for raw and analysed data and materials will be promptly reviewed by McGill University to verify if the request is subject to any intellectual property or confidentiality obligations. Anonymized data will be shared upon request to the study’s senior author from a qualified academic investigator for the sole purpose of replicating the procedures and results presented in this article. Any data and materials that can be shared will be released via a material transfer agreement. Data are not publicly available due to information that could compromise the privacy of research participants. Related documents, including study protocol and informed consent forms, can similarly be made available upon request.
