# Supplementary material for: Predicting functional decline in aging and Alzheimer’s disease with PET-based Braak staging
Source: Brain Commun. 2024 Feb 26;6(2):fcae043. doi: 10.1093/braincomms/fcae043 (PMC10935644; doi:10.1093/braincomms/fcae043)
Supplement: fcae043_Supplementary_Data [file fcae043_supplementary_data.pdf]

Supplementary Table 1 Demographic, clinical and biomarkers characteristics of the participants with a follow-up visit

	Braak 0 (N = 73)	Braak I/II (N = 58)	Braak III/IV (N = 21)	Braak V/VI (N = 36)	p-value
**Age (years), mean (s.d.)**	73.03 (6.3)	74.50 (5.8)	75.58 (4.6)	69.96 (6.9)	**<0.01**
**Female, n (%)**	45 (61.6%)	40 (69.0%)	12 (57.1%)	22 (61.1%)	0.73
**Years of education, mean (s.d.)**	15.73 (3.6)	14.97 (3.5)	15.62 (3.2)	14.68 (3.6)	0.40
**Clinical diagnosis, n (%)**	71 CU (97.3%)<br>2 MCI (2.7%)	42 CU (72.4%)<br>15 MCI (25.9%)<br>1 AD (1.7%)	12 CU (57.1%)<br>5 MCI (23.8%)<br>4 AD (19.0%)	13 MCI (36.1%)<br>23 AD (63.9%)	**<0.001**
**MMSE, mean (s.d.)**	29.10 (1.1)	28.81 (1.5)	28.95 (1.6)	24.22 (4.2)	**<0.001**
**APOE ε4 carriers, n (%)**	17 (23.3%)	18 (31.0%)	9 (42.9%)	21 (58.3%)	**0.02**
**Neocortical [^{18}F]AZD4694 SUVR, mean (s.d.)**	1.36 (0.2)	1.71 (0.5)	1.91 (0.6)	2.54 (0.4)	**<0.001**
**Follow-up time (years), mean (s.d.)[a]**	2.01 (0.7)	2.02 (0.7)	1.98 (0.6)	1.79 (0.7)	0.50

p-values were calculated using the Kruskal-Wallis test for age, years of education, MMSE, and SUVRs. Chi-squared test was performed for sex proportion and APOE ε4 status comparison. Statistical significance was considered if p<0.05 (results in bold). Legend: AD: Alzheimer's disease; CU: cognitively unimpaired; MCI: mild cognitive impairment; MMSE: Mini-Mental State Examination; ROI: region of interest; s.d.: standard deviation; SUVR: standardized uptake value ratio.

[a] 3 missing data

Supplementary Table 2. Regression coefficients of the association between baseline PET-based Braak stages and the annual change in the functional scores without the exclusion of outliers

	FAQ			Ecog			Functional CDR-SB		
	Beta (95% CI)	T-value	p-value	Beta (95% CI)	T-value	p-value	Beta (95% CI)	T-value	p-value
**PET-based Braak stage I-II**	0.08 (-0.37 – 0.52)	0.35	0.73	0.05 (-0.35 – 0.44)	0.24	0.81	-0.04 (-0.34 – 0.26)	-0.26	0.79
**PET-base Braak stage III-IV**	-0.08 (-0.68 – 0.52)	-0.27	0.79	0.07 (-0.47 – 0.62)	0.27	0.79	-0.03 (-0.45 – 0.40)	-0.12	0.91
**PET-based Braak stage V-VI**	1.23 (0.44 – 2.02)	3.08	**0.003**	1.06 (0.42 – 1.70)	3.29	**0.001**	1.29 (0.79 - 1.78)	5.17	**<0.0001**
**Age (years)**	-0.15 (-0.40 – 0.10)	-1.21	0.23	0.09 (-0.12 – 0.31)	0.85	0.39	-0.14 (-0.29 – 0.01)	-1.80	0.07
**Sex (male)**	0.04 (-0.33 – 0.41)	0.22	0.83	0.08 (-0.25 – 0.41)	0.48	0.64	0.02 (-0.23 – 0.26)	0.12	0.90
**Neocortical [^{18}F]AZD4694 SUVR**	0.09 (-0.22 – 0.39)	0.57	0.57	0.26 (0.007 – 0.50)	2.04	**0.04**	0.15 (-0.05 – 0.34)	1.50	0.14

Adjusted R^2: 0.50, F stat: 17.61 (FAQ); Adjusted R^2: 0.26, F stat: 8.36 (ECog); Adjusted R2: 0.36, F stat: 18.15 (Functional CDR-SB). Statistical significance was considered if $p<0.05$ (results in bold). Legend: CDR-SB: clinical dementia rating sum of boxes; ECog: everyday cognition; FAQ: functional activities questionnaire; PET: positron emission tomography.

Supplementary Table 3 Regression coefficients of the cross-sectional association between PET-based Braak stages and functional scores at baseline in models adjusting for clinical diagnoses

	FAQ			Ecog			Functional CDR-SB		
	Beta (95% CI)	T-value	p-value	Beta (95% CI)	T-value	p-value	Beta (95% CI)	T-value	p-value
**PET-based Braak stage I-II**	-0.07 (-0.30 – 0.15)	-0.65	0.51	-0.02 (-0.25 – 0.21)	-0.20	0.84	0.02 (-0.20 – 0.24)	0.17	0.87
**PET-base Braak stage III-IV**	0.03 (-0.27 – 0.33)	0.20	0.84	0.04 (-0.27 – 0.35)	0.28	0.78	-0.09 (-0.39 – 0.21)	-0.60	0.55
**PET-based Braak stage V-VI**	0.23 (-0.15 – 0.62)	1.20	0.23	0.29 (-0.10 – 0.69)	1.47	0.14	0.33 (-0.05 - 0.71)	1.73	0.09
**Age (years)**	-0.03 (-0.12 – 0.07)	-0.57	0.57	-0.05 (-0.15 – 0.04)	-1.06	0.29	-0.02 (-0.11 – 0.06)	-0.57	0.57
**Sex (male)**	0.02 (-0.16 – 0.19)	0.19	0.85	0.06 (-0.12 – 0.24)	0.66	0.51	0.04 (-0.13 – 0.21)	0.45	0.65
**Neocortical [^{18}F]AZD4694 SUVR**	-0.003 (-0.16 – 0.15)	-0.04	0.97	0.07 (-0.09 – 0.23)	0.86	0.39	0.08 (-0.06 – 0.21)	1.12	0.26
**Clinical diagnosis (MCI)**	0.35 (0.04 – 0.66)	2.26	**0.02**	0.55 (0.23 – 0.86)	3.42	**<0.001**	-0.01 (-0.30 – 0.28)	-0.09	0.93
**Clinical diagnosis (AD dementia)**	2.18 (1.80 – 2.57)	11.17	**<0.0001**	1.91 (1.52 – 2.31)	9.58	**<0.0001**	1.52 (1.15 – 1.90)	7.98	**<0.0001**

Adjusted R^2: 0.62, F stat: 45.69 (FAQ); Adjusted R^2: 0.60, F stat: 41.66 (ECog); Adjusted R^2: 0.53, F stat: 41.37 (Functional CDR-SB). Statistical significance was considered if $p<0.05$ (results in bold). Legend: CDR-SB: clinical dementia rating sum of boxes; ECog: everyday cognition; FAQ: functional activities questionnaire; PET: positron emission tomography.

Supplementary Table 4 Regression coefficients of the association between baseline PET-based Braak stages and the annual change in the functional scores in models adjusting for clinical diagnoses

	FAQ			Ecog			Functional CDR-SB		
	Beta (95% CI)	T-value	p-value	Beta (95% CI)	T-value	p-value	Beta (95% CI)	T-value	p-value
**PET-based Braak stage I-II**	-0.04 (-0.38 – 0.30)	-0.24	0.81	-0.06 (-0.44 – 0.33)	-0.29	0.77	-0.02 (-0.30 – 0.26)	-0.15	0.88
**PET-base Braak stage III-IV**	-0.08 (-0.55 – 0.40)	-0.32	0.75	-0.14 (-0.67 – 0.39)	-0.51	0.61	-0.22 (-0.62 – 0.17)	-1.10	0.27
**PET-based Braak stage V-VI**	1.28 (0.63 – 1.93)	3.90	**<0.001**	0.49 (-0.21 – 1.18)	1.38	0.17	0.65 (0.13 - 1.17)	2.47	**0.01**
**Age (years)**	0.01 (-0.18 – 0.21)	0.15	0.88	0.13 (-0.08 – 0.34)	1.20	0.23	-0.08 (-0.22 – 0.05)	-1.20	0.23
**Sex (male)**	-0.02 (-0.30 – 0.27)	-0.13	0.90	0.13 (-0.19 – 0.45)	0.78	0.43	0.02 (-0.21 – 0.24)	0.14	0.89
**Neocortical [^{18}F]AZD4694 SUVR**	-0.03 (-0.32 – 0.25)	-0.21	0.83	0.06 (-0.23 – 0.34)	0.40	0.69	0.12 (-0.09 – 0.34)	1.16	0.25
**Clinical diagnosis (MCI)**	0.56 (0.02 – 1.10)	2.06	**0.04**	0.47 (-0.10 – 1.03)	1.64	0.10	-0.16 (-0.56 – 0.25)	-0.76	0.45
**Clinical diagnosis (AD dementia)**	1.36 (0.60 – 2.12)	3.55	**<0.001**	1.37 (0.58 – 2.16)	3.45	**<0.001**	1.21 (0.67 – 1.75)	4.43	**<0.0001**

Adjusted R^2: 0.56, F stat: 16.32 (FAQ); Adjusted R^2: 0.33, F stat: 8.38 (ECog); Adjusted R^2: 0.48, F stat: 21.05 (Functional CDR-SB). Statistical significance was considered if $p<0.05$ (results in bold). Legend: CDR-SB: clinical dementia rating sum of boxes; ECog: everyday cognition; FAQ: functional activities questionnaire; PET: positron emission tomography.
